# A systematic analysis of the global and regional burden of colon and rectum cancer and the difference between early- and late-onset CRC from 1990 to 2019

**DOI:** 10.3389/fonc.2023.1102673

**Published:** 2023-02-15

**Authors:** Liu-Bo Li, Li-Yu Wang, Da-Ming Chen, Ying-Xia Liu, Yuan-Hui Zhang, Wei-Xiang Song, Xu-Bo Shen, Sheng-Quan Fang, Zheng-Yuan Ma

**Affiliations:** ^1^ Yueyang Hospital of Integrated Traditional Chinese and Western Medicine Affiliated to Shanghai University of Traditional Chinese Medicine, Shanghai, China; ^2^ Shanghai Information Center for Life Sciences, Shanghai Institute of Nutrition and Health, University of Chinese Academy of Sciences, Chinese Academy of Sciences, Shanghai, China

**Keywords:** colorectal cancer, the global burden, estimated annual percentage changes, Hdi (human development index), age-standardised incidence rate

## Abstract

The burden of colorectal cancer (CRC) varies substantially across different geographical locations. However, there was no further quantitative analysis of regional social development and the disease burden of CRC. In addition, the incidence of early- and late-onset CRC has increased rapidly in developed and developing regions. The main purpose of this study was to investigate the trends in CRC burden across different regions, in addition to the epidemiological differences between early and late-onset CRC and their risk factors. In this study, estimated annual percentage change (EAPC) was employed to quantify trends in age-standardized incidence rate (ASIR), mortality rate, and disability-adjusted life-years. Restricted cubic spline models were fitted to quantitatively analyze the relationship between trends in ASIR and Human Development Index (HDI). In addition, the epidemiological characteristics of early- and late-onset CRC were investigated using analyses stratified by age groups and regions. Specifically, meat consumption and antibiotic use were included to explore the differences in the risk factors for early- and late-onset CRC. The quantitative analysis showed that the ASIR of CRC was exponentially and positively correlated with the 2019 HDI in different regions. In addition, the growing trend of ASIR in recent years varied substantially across HDI regions. Specifically, the ASIR of CRC showed a significant increase in developing countries, while it remained stable or decreased in developed countries. Moreover, a linear correlation was found between the ASIR of CRC and meat consumption in different regions, especially in developing countries. Furthermore, a similar correlation was found between the ASIR and antibiotic use in all age groups, with different correlation coefficients for early-onset and late-onset CRC. It is worth mentioning that the early onset of CRC could be attributable to the unrestrained use of antibiotics among young people in developed countries. In summary, for better prevention and control of CRC, governments should pay attention to advocate self-testing and hospital visits among all age groups, especially among young people at high risk of CRC, and strictly control meat consumption and the usage of antibiotics.

## Introduction

Estimates from the International Agency for Research on Cancer in 2020 suggested that, globally, colorectal cancer (CRC) constituted over 1.9 million new cases and 900,000 deaths annually (1), ranking the third most commonly diagnosed malignancy and the second leading cause of cancer death (2). Meanwhile, CRC resulted in 19.0 million disability-adjusted life-years (DALYs) globally, with an age-standardized rate (ASR) of 235.7 DALYs per 100,000 person-years (3). In addition, CRC showed obvious regional and economic differences (4), which resulted in significant geographic heterogeneity in the age-standardized incidence rate (ASIR), mortality rate (ASMR), and DALYs rate (ASDR). As such, CRC accounted for a higher proportion of cancer burden and gradually surpassed infection-related cancers in the regions with high Human Development Index (HDI) (5), while ASIR showed a stable and even downward trend (6). However, the incidence of early-onset CRC (usually defined as CRC patients younger than 50 years old) has gradually been increasing since the mid-1990s for unknown reasons (7) in developed countries (8, 9). Meanwhile, the incidence of late-onset CRC (usually defined as CRC patients older than 50 years old) has increased sharply in developing countries due to the industrialization, the widespread acceptance of unhealthy lifestyles and diets, and environmental deterioration (10).

Therefore, it is necessary to conduct a comprehensive study on economic development and the CRC burden to explain the geographic heterogeneity. In addition, there is a need for in-depth epidemiological research on CRC burden in different regions. Meanwhile, evidence on the risk factors for early- and late-onset CRC is also needed. The contributions of the risk factors of CRC varied with time and by geographical region. For example, smoking and alcohol use have gradually decreased with the implementation of anti-smoking and alcohol policies and campaigns (11–13). In contrast, the influence of diet patterns, especially meat consumption, is affected by economic development and vary widely globally. In addition, antibiotic use was thought to be related to the incidence of early-onset CRC (14). This study investigated these two risk factors in early- and late-onset CRC.

As a non-communicable disease, reducing the global burden of CRC will help to achieve the Sustainable Development Goals (SDGs) of reducing by one-third premature mortality from non-communicable diseases by 2030 (15, 16). The Global Burden of Diseases (GBD), Injuries, and Risk Factors Study 2019 assessed the CRC burden in 195 countries and territories globally, providing a unique perspective to help understand the general situation and time-varying landscape of CRC. Based on the 2019 report of the GBD Colorectal Cancer Collaborators (17), this study focused on the trends in ASR using estimated annual percentage changes (EAPCs) (18) from 1990 to 2019. In particular, to elucidate the underlying reasons for the geographic heterogeneity of CRC burden, regression analysis and restricted cubic spline analysis (RCS) (19) were implemented to analyze the relationship between HDI and EAPC in ASR. In addition, this study analyzed EAPCs in ASR across different age groups and regions and visually showed differences in the burden of early- and late-onset CRC. Furthermore, the correlation analysis of ASIR between dietary structure changes (represented by global meat consumption) and potential risk factors (represented by antimicrobial usage) was used to explore potential risk factors for early-onset and late-onset CRC. As an extension and complementary study, this analysis could potentially promote improvements in CRC healthcare and clinical guidelines.

## Materials and methods

### Data source

Data on incidence, mortality, DALYs, age-standardized incidence rate (ASIR), age-standardized mortality rate (ASMR), age-standardized DALYs rate (ASDR), and risk exposures of CRC were obtained from the Global Burden of Disease Study 2019 (GBD 2019) on the GHDx (Global Health Data Exchange) data source (http://ghdx.healthdata.org/gbd-results-tool). Risk exposures, including behavioral factors (alcohol use and smoking), dietary factors (including high fasting plasma glucose, diet low in calcium, diet low in milk, diet low in fiber, diet high in red meat, and diet high in processed meat), and metabolic factors (including high fasting plasma glucose and high body-mass index) were included in this study.

According to geographical features, the world was divided into 21 geographic regions, including Australasia, East Asia, and Eastern Europe. Based on GBD 2019 data, countries and territories were categorized into five regions based on the socio-demographic index (SDI), including high, high-middle, middle, low-middle, and low SDI. SDI reflects the level of medical health. Meanwhile, comprised by macro indicators of human development, the Human Development Index (HDI) was obtained from the United Nations Development Program (UNDP; http://hdr.undp.org/en/data). The relationship between human development and disease burden was investigated using correlation analysis of GBD data and HDI. Trends in disease burden were assessed across different age groups (10–14, 15–19, 20–24, 25–29, 30–34, 35–39, 40–44, 45–49, 50–54, 55–59, 60–64, 65–69, 70–74, 75–79, 80–84, 85–89, 90–94, 95 plus). In addition, this study used data on antibiotic use (https://www.tropicalmedicine.ox.ac.uk/research/oxford/microbe/gram-project/antibiotic-usage-and-consumption) and data on the economic statistics on the nutrition and health industry-global meat consumption per capita from 1990 to 2029 (https://www.ckcest.cn/default/es3/detail/4004/dw_dataset/C9A75565E5D00001E17C1C012A571CE2).

### Statistical analysis

EAPC was used to quantify trends in ASRs, calculated using a generalized linear model with a Gaussian distribution. A regression line was used to estimate the natural logarithm of the rates; for example, y = α + βx + ϵ, where y = ln(ASR) and x = calendar year. The EAPC was calculated as 100 × [exp(β)-1] alongside the 95% confidence interval (CI) using linear regression (18). An increasing trend was observed when the EAPC value and its 95% CI were larger than 0. In contrast, a decreasing trend was observed when the EAPC value and its 95% CI were less than 0. In addition, a correlation analysis between HDI and disease burden was analyzed using Pearson’s correlation coefficient. Regression analysis and restricted cubic spline models (RCS) models were fitted to explore a nonlinear relationship between the 2019 HDI and EAPC in ASR. Finally, Pearson’s correlation analysis was used to analyze the relationship of ASIR with global meat consumption and antimicrobial usage. All statistics were performed using R (Version 3.6.0). A p-value of less than 0.05 was considered statistically significant.

### Patient and public involvement

Patients or the public were not involved in the design, data collection, analyses, or interpretation of this research.

## Results

### Trends in the incidence of CRC

**Figure 1:**
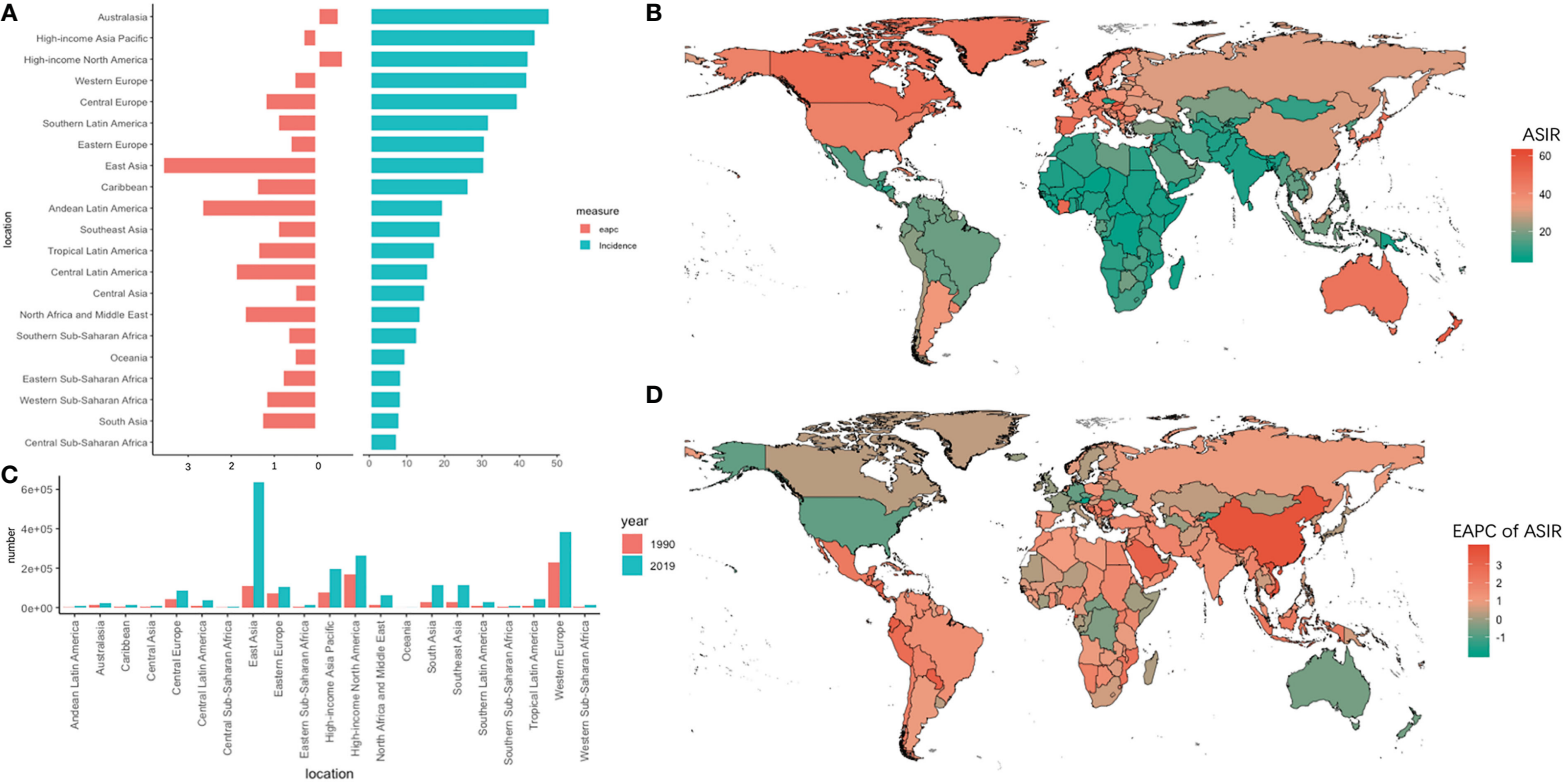
The absolute number of new cases and age-standardized rate of incidence and its EAPC value in 195 countries/territories and 21 regions. **(A)** the ASIR and its corresponding EAPC value in 21 regions of the world in 2019, in which red bar chart represents EAPC, blue bar chart represents ASIR; **(B)** ASIR of CRC in 195 countries/territories in 2019; **(C)** The absolute number of new cases in 21 regions in 1990 and 2019; **(D)** The EAPC of ASIR in 195 countries/territories in 2019.

Globally, the absolute number of colorectal new cancer cases reached 2166.17 × 10^3^ (95% UI = 1996.29 × 10^3^ to 2342.84 × 10^3^) in 2019, representing a 1.57 times increase from 842.10 × 10^3^ (95% UI = 810.41 × 10^3^ to 868.57 × 10^3^) in 1990. The ASIR increased gradually (EAPC = 0.58, 95% UI = 0.52–0.65) from 22.25 (95% UI = 21.30–22.97) per 100,000 persons in 1990 to 26.71 (95% UI = 24.58–28.89) per 100,000 persons in 2019 (**Table 1**). The largest increasing trend occurred in the middle SDI region (EAPC = 2.61, 95% UI = 2.44 to 2.77, **Figure 1**).

**Table 1:** The incident cases and age-standardized incidence of CRC in 1990 and 2019, and its temporal trends from 1990 to 2019.

Characteristics	1990		2019		1990–2019
	Incident cases No. x10^3^ (95% UI)	ASR per 100,000 No. (95% UI)	Incident cases No. x10^3^ (95% UI)	ASR per 100,000 No. (95% UI)	EAPC No. (95% CI)
Overall	842.1 (810.41–868.57)	22.25 (21.30–22.97)	2166.17 (1996.29–2342.84)	26.71 (24.58–28.89)	0.58 (0.52–0.65)
Sex					
Male	428.21 (413.48–444.29)	25.19 (24.24–26.11)	1239.73 (1133.17–1359.15)	33.06 (30.22–36.15)	0.95 (0.87–1.02)
Female	413.89 (393.4–432.7)	19.92 (18.83–20.81)	926.43 (831.86–1011.62)	21.21 (19.04–23.16)	0.11 (0.03–0.18)
Socio-demographic index					
Low	14.28 (11.96–16.83)	6.2 (5.19–7.26)	36.95 (32.89–40.03)	7.33 (6.55–8.13)	0.81 (0.69–0.93)
Low-middle	40.77 (36.76–45.65)	6.87 (6.23–7.71)	151.95 (137.8–166.92)	11.27 (10.23–12.35)	1.74(1.69–1.78)
Middle	104.83 (97.28–113.06)	10.28 (9.48–11.03)	465.98 (418.6–465.98)	18.87 (16.98–20.86)	2.61 (2.44–2.77)
Middle-high	237.9 (229.79–249.1)	22.6 (21.75–23.36)	655.82 (594.86–716.67)	32.4 (29.4–35.39)	1.29 (1.19–1.4)
High	443.89 (427.71–453.34)	42.45 (40.91–43.35)	798.58 (715.63–873.25)	42.77 (38.75–46.64)	-0.17 (-0.28 to -0.06)
Region					
Asia Pacific-high income	71.18 (73.96–79.22)	38.69 (36.92–39.76)	196.37 (166.42–225.64)	44.58 (38.38–51.09)	0.35 (0.23–0.47)
Central Asia	6.75 (6.52–6.98)	13.99 (13.51–14.46)	10.95 (9.99–12.01)	15.21 (13.93–16.61)	0.54 (0.33–0.76)
East Asia	112.32 (100.31–125.56)	12.77 (11.44–14.26)	637.1 (548.9–738.55)	30.94 (26.75–35.74)	3.61 (3.33–3.89)
South Asia	29.94 (26.44–34.06)	5.44 (4.79–6.21)	113.71 (98.19–129.53)	8.31 (7.21–9.43)	1.31 (1.18–1.44)
Southeast Asia	27.9 (24.57–30.66)	10.81 (9.6–11.81)	117.01 (96.63–136.24)	19.3 (15.97–22.4)	1.93 (1.87–1.98)
Australasia	12.03 (11.52–12.42)	51.57 (49.36–53.25)	23.67 (19.44–28.85)	48.34 (39.6–59.06)	-0.52 (-0.65 to -0.39)
Caribbean	4.69 (4.49–4.85)	18.19 (17.34–18.83)	13.81 (11.81–15.96)	26.73 (22.86–30.86)	1.43 (1.37–1.49)
Central Europe	41.59 (40.37–42.59)	28.4 (27.5–29.11)	84.47 (74.55–95.45)	30.87 (35.19–45.07)	1.23 (1.11–1.35)
Eastern Europe	70.4 (68.29–72.72)	25.09 (24.32–25.92)	106.12 (96.25–117.07)	31.11 (28.22–34.37)	0.56 (0.4–0.73)
Western Europe	229.47 (220.38–234.89)	39.57 (38.03–40.47)	382.44 (332.8–432.45)	42.42 (37.09–48.26)	0.04 (-0.17 to 0.24)
Andean Latin America	2.02 (1.8–2.24)	9.99 (8.85–11.07)	11.1 (8.93–13.47)	19.95 (16.07–24.18)	2.70 (2.52–2.88)
Central Latin America	7.48 (7.2–7.66)	9 (8.59–9.26)	37.54 (32.21–43.87)	15.93 (13.67–18.62)	1.92 (1.83–2.02)
Southern Latin America	10.93 (10.52–11.26)	22.41 (23.07–24.82)	28.87 (21.48–33.61)	32.22 (25.69–40.39)	0.94 (0.83–1.05)
Tropical Latin America	10.71 (10.34–11.05)	12 (11.46–12.39)	42.9 (40.12–44.93)	17.76 (16.58–18.63)	1.4 (1.23–1.65)
North Africa and Middle East	15.42 (12.97–18.18)	4.47 (3.76–5.27)	60 (53.35–67.56)	13.93 (12.32–15.6)	1.71 (1.50–1.91)
North America-high income	167.9 (160.79–172.26)	47.46 (45.59–48.63)	260.91 (229.91–295.69)	42.71 (37.61–48.61)	-0.62 (-0.72 to -0.51)
Oceania	0.25 (0.19–0.3)	8.34 (6.62–9.95)	0.69 (0.56–0.86)	9.99 (8.16–12.13)	0.55 (0.49–0.62)
Central Sub-Saharan Africa	1.61 (1.25–2.04)	7.42 (5.91–9.27)	3.96 (3.01–5.11)	7.68 (5.92–10.07)	0.06 (-0.2 to 0.32)
Eastern Sub-Saharan Africa	5.2 (4.34–6.14)	7.01 (5.83–8.25)	14.22 (12.13–16.89)	8.83 (7.64–10.37)	0.83 (0.75–0.91)
Southern Sub-Saharan Africa	2.87 (2.5–3.34)	10.73 (9.26–12.71)	7.1 (6.39–7.88)	13.07 (11.8–14.45)	0.70 (0.53–0.88)
Western Sub-Saharan Africa	5.43 (4.4–6.64)	6.55 (5.35–7.95)	15.32 (12.89–17.82)	8.72 (7.45–10.03)	1.21 (1.1–1.32)

The absolute number of new cases increased by 4.67 times in 30 years, and the ASIR increased fastest in East Asia (EAPC = 3.61, 95% UI = 3.33–3.89). The ASIR overran 40 per 10,000 persons in the Asia Pacific-high income, Australasia, Western Europe, and North America-high income regions. Among them, ASIR declined the most (EAPC = -0.62, 95% UI = -0.72 to -0.51) in the North America-high income region. In Africa, the overall incidence rate showed a slowly growing trend, with the highest growth trend in Western sub-Saharan Africa (EAPC = 1.21, 95% UI = 1.10 to 1.32). In particular, the EAPC exceeded 3.6 in Equatorial Guinea, Vietnam, and China.

### Trends in mortality due to CRC

In 2019, 1085.80 (95% UI 1002.80 to 1149.68) × 10^3^ deaths were attributed to CRC (**Table 2**), corresponding to a 109.56% (95% UI = 96.20% to 121.74%) increase compared to 1990. In addition, the ASMR showed a decreasing trend during the last three decades (EAPC = -0.21, 95% UI = -0.28 to -0.14). Meanwhile, the high SDI region showed a downward trend (EAPC=-1.09, 95% UI= -1.17 to -1.00), while the middle and low-middle regions showed obvious increasing trends (EAPC = 1.24, 95% UI = 1.10–1.37; EAPC = 1.15, 95% UI = 1.11–1.19, respectively).

**Table 2:** The cases and age-standardized of deaths and DALYs of CRC in 1990 and 2019, and its temporal trends from 1990 to 2019.

Characteristics	No. of deaths			DALYs		
	Number in 2019, cases X 10^3^ (95% UI)	Percentage change in absolute number (%)	EAPC (95% CI)	Number in 2019, cases X 10^3^ (95% UI)	Percentage change in absolute number (%)	EAPC
						(95%CI)
Total	1085.80 (1002.80–1149.68)	109.56 (96.20–121.74)	-0.21 (-0.28 to -0.14)	24,284.09 (22,614.92–25,723.22)	95.71 (82.15–108.59)	-0.21 (-0.26 to -0.15)
Sex						
Male	594.18 (550.18–638.03)	130.92 (110.50–150.51)	0.1 (0.03–0.17)	13,959.59 (12,859.72–15,045.72)	115.64 (93.77–136.18)	0.13 (0.06–0.2)
Female	491.62 (437.55–532.38)	88.50 (74.84–102.42)	-0.59 (-0.67 to -0.52)	10,324.5 (9494.91–11,149.97)	73.79 (60.22–87.63)	-0.65 (-0.71 to -0.6)
SDI						
Low	34.66 (30.96–38.61)	157.80 (112.08–210.23)	0.58 (0.52–0.64)	942.42 (835.79–1059.27)	147.32 (101.56–200.78)	0.44 (0.38–0.5)
Low-middle	116.55 (105.51–128.33)	223.28 (171.75–267.87)	1.15 (1.11–1.19)	2998.93 (2703.93–3314.97)	189.96 (171.75–267.87)	1.01 (0.98–1.05)
Middle	279.78 (251.15–306.14)	235.57 (196.69–275.09)	1.24 (1.1–1.37)	6990.43 (6308.90–7671.29)	195.47 (159.84–275.09)	1.11 (0.99–1.24)
High-middle	326.64 (299.66–349.53)	100.78 (85.89–115.23)	-0.04 (-0.15 to 0.06)	7174.86 (6649.07–7693.26)	81.16 (67.40–95.16)	-0.18 (-0.28 to -0.08)
High	327.57 (294.90–345.58)	47.35 (39.21–52.76)	-1.09 (-1.17 to -1.00)	6164.66 (5754.65–6435.90)	32.28 (27.55–36.63)	-1.11 (-1.17 to -1.04)
Regions						
East Asia	275.60 (238.24–317.89)	230.83 (174.13–295.52)	1.4 (1.17–1.64)	6712.86 (5774.28–7735.91)	181.40 (132.43–238.11)	1.24 (1.02–1.46)
South Asia	94.85 (81.52–109.07)	247.31 (177.91–314.50)	0.91 (0.79–1.04)	2419.10 (2078.02–2782.57)	207.50 (146.30–267.01)	0.8 (0.68–0.92)
Southeast Asia	82.02 (67.62–94.61)	246.99 (189.60–300.06)	1.25 (1.19–1.31)	2142.43 (1780.49–2482.29)	218.40 (166.60–265.82)	1.12 (1.05–1.18)
Central Asia	7.47 (6.82–8.17)	45.80 (33.65–60.40)	0.33 (0.17–0.5)	199.84 (182.01–219.94)	35.20 (23.23–49.85)	-0.37 (-0.53 to -0.21)
High-income Asia Pacific	76.93 (64.82–83.6)	124.03 (98.74–138.23)	-0.68 (-0.75 to -0.62)	1327.82 (1186.12–1414.81)	64.65 (52.3–72.59)	-0.88 (-0.96 to -0.8)
Oceania	0.55 (0.44–0.68)	167.41 (230.06–114.82)	0.41 (0.34–0.49)	16.31 (12.93–20.56)	162.99 (108.35–230.10)	0.36 (0.3–0.42)
Australasia	8.38 (7.57–8.98)	48.65 (38.89–57.93)	-1.73 (-1.88 to -1.59)	163.25 (150.87–173.96)	29.41 (22.03–37.23)	-1.89 (-2.03 to -1.17)
Eastern Europe	63.48 (57.18–70.01)	27.39 (15.58–39.68)	-0.31 (-0.51 to -0.12)	1419.10 (1287.54–1571.37)	15.25 (4.68–27.06)	-0.57 (-0.79 to -0.34)
Western Europe	172.45 (155.34–181.81)	36.74 (25.02–36.67)	-1.09 (-1.25 to -0.92)	3008.23 (2815.06–3152.89)	16.01 (11.24–20.10)	-1.17 (-1.3 to -1.03)
Central Europe	51.57 (45.64–57.75)	67.28 (48.95–85.76)	0.32 (0.21–0.42)	1052.15 (922.92–1184.25)	46.84 (29.53–64.72)	0.17 (0.07–0.27)
High-income North America	95.66 (88.32–99.69)	33.04 (28.93–36.55)	-1.22 (-1.32 to -1.13)	1987.11 (1895.87–2059.77)	31.74 (28.13–35.37)	-1.07 (-1.16 to -0.97)
Andean Latin America	5.63 (4.59–6.79)	269.75 (201.86–349.77)	1.16 (1.02–1.3)	125.58 (101.75–151.80)	230.17 (163.34–321.74)	1.04 (0.89–1.18)
Central Latin America	22.47 (19.54–26.00)	292.01 (241.04–349.36)	0.97 (0.93–1.01)	539.64 (465.2–627.07)	264.23 (214.09–322.09)	1.11 (1.07–1.15)
Caribbean	7.99 (6.94–9.18)	143.26 (112.23–175.87)	0.63 (0.57–0.68)	172.02 (147.19–200.17)	126.54 (94.75–160.28)	0.6 (0.54–0.66)
Tropical Latin America	27.7 (25.67–29.09)	226.92 (208.22–241.92)	0.60 (0.44–0.75)	660.13 (625.56–687.74)	197.23 (182.41–211.04)	0.68 (0.51–0.85)
Southern Latin America	17.93 (16.77–18.97)	103.09 (92.66–114.21)	0.15 (0.05–0.25)	366.44 (347.73–385.44)	87.63 (77.90–97.75)	0.17 (0.09–0.24)
Eastern Sub-Saharan Africa	12.72 (10.94–15.00)	159.54 (109.72–221.98)	0.69 (0.62–0.76)	356.43 (301.93–425.61)	154.30 (101.54–225.87)	0.54 (0.47–0.62)
Southern Sub-Saharan Africa	5.92 (5.33–6.58)	130.50 (105.16–168.87)	0.45 (0.24–0.66)	147.78 (132.44–165.54)	125.01 (98.84–158.74)	0.49 (0.28–0.70)
Western Sub-Saharan Africa	13.77 (11.70–16.07)	165.41 (117.27–232.00)	1.05 (0.95–1.15)	353.24 (295.57–420.70)	165.74 (113.86–238.56)	0.88 (0.79–0.97)
North Africa and Middle East	39.15 (34.76–44.11)	199.32 (140.51–280.08)	0.81 (0.62–1.00)	1013.63 (896.16–1146.53)	177.60 (120.11–249.96)	0.56 (0.38–0.74)
Central Sub-Saharan Africa	3.54 (2.7–4.61)	133.84 (60.65–233.68)	-0.11 (-0.36 to 0.14)	100.99 (75.75–131.45)	131.68 (58.49–224.76)	-0.14 (-0.38 to 0.10)

**Figure 2:**
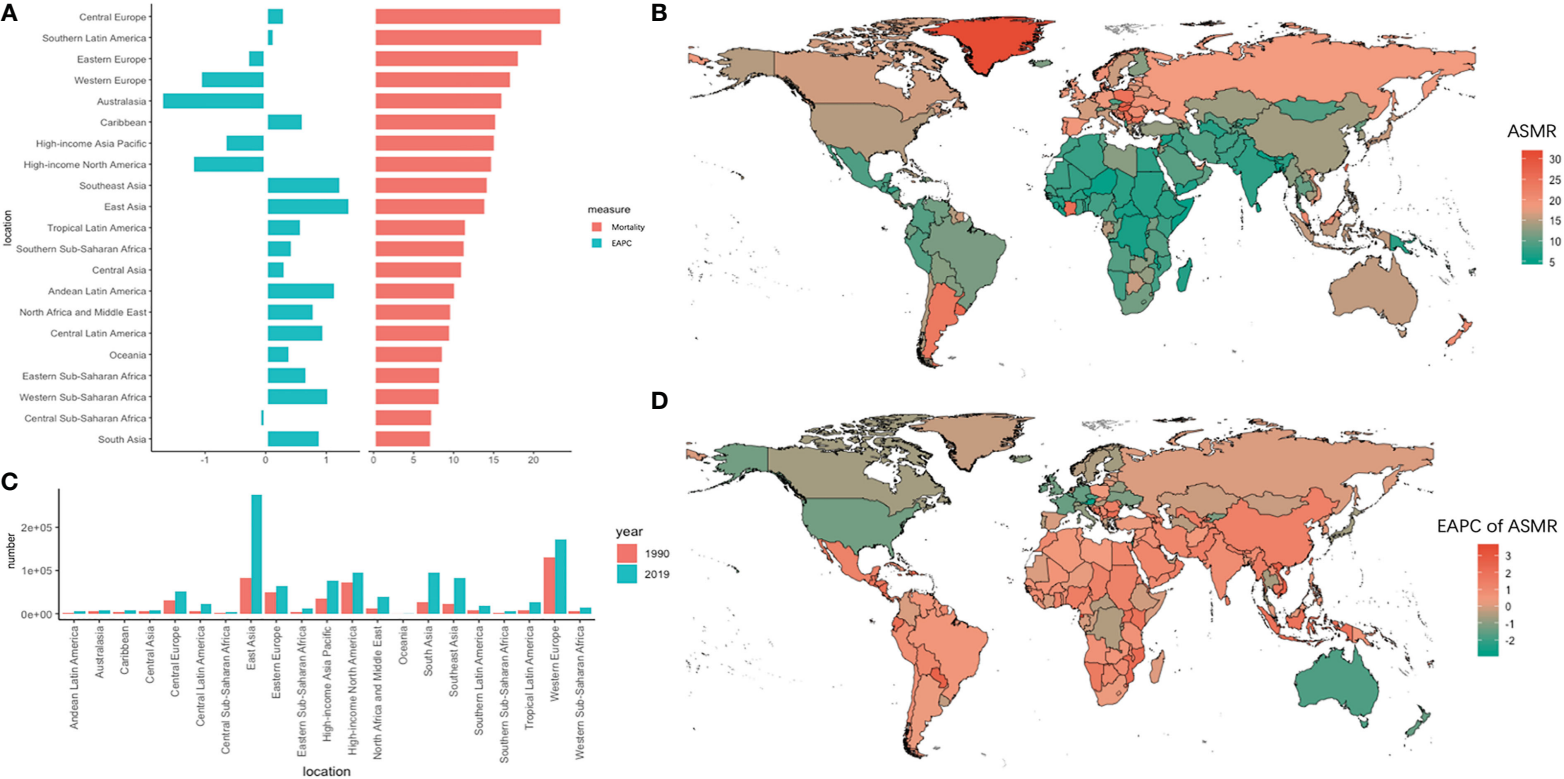
The absolute number of deaths and age-standardized rate of mortality and its EAPC value in 195 countries/territories and 21 regions. **(A)** the ASMR and its corresponding EAPC value in 21 regions of the world in 2019, in which red bar chart represents ASMR, blue bar chart represents EAPC of ASMR; **(B)** ASMR of CRC in 195 countries/territories in 2019; **(C)** The absolute number of deaths in 21 regions in 1990 and 2019; **(D)** The EAPC of ASMR in 195 countries/territories in 2019.

The absolute new number of deaths in East Asia increased by 2.31 times, with 275.60 (UI = 238.24–317.89) × 10^3^ deaths in 2019 and showed the highest increase in the trend for ASMR (EAPC = 1.4, 95% UI = 1.17–1.64) within 30 years. On the contrary, Australasia showed the lowest decrease in ASMR (EAPC = -1.73, 95% UI = -1.88 to -1.59). Meanwhile, in Africa, Western sub-Saharan Africa showed the highest increase trend (EAPC = 1.05, 95% UI = 0.95–1.15). Among the 195 countries and territories, China had the highest absolute number of new deaths in 2019 (261,776 new cases), followed by the United States, India, and Japan. Meanwhile, Austria and Singapore had the fastest decline in ASMR within 30 years, with EAPCs of -3.09 and -2.32, respectively (**Figure 2**).

### Trends in DALYs due to CRC

**Figure 3:**
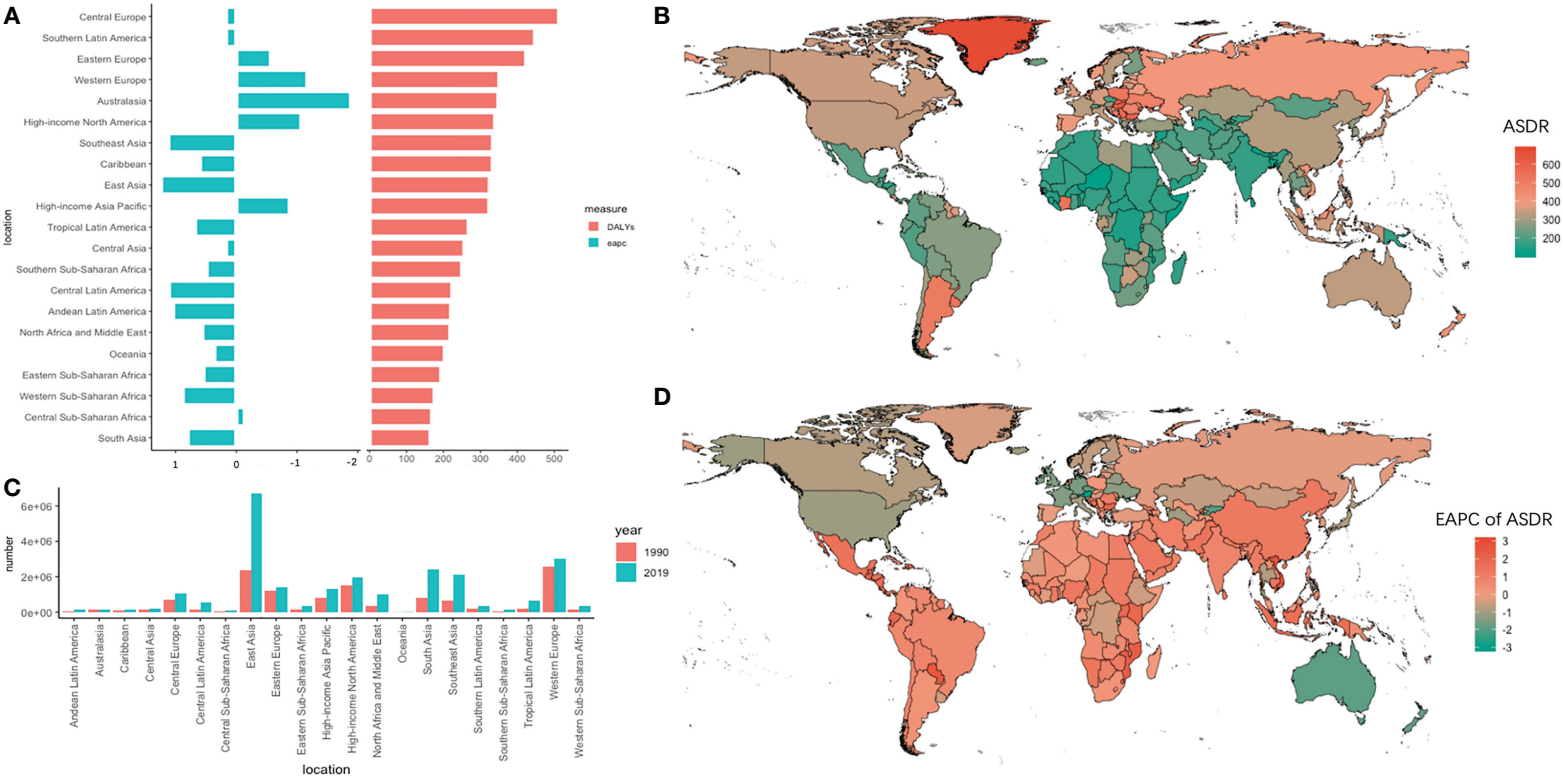
The absolute number of DALYs and age-standardized rate of DALYs and its EAPC value in 195 countries/territories and 21 regions. **(A)** the ASDR and its corresponding EAPC value in 21 regions of the world in 2019, in which red bar chart represents ASDR, blue bar chart represents EAPC of ASDR; **(B)** ASDR of CRC in 195 countries/territories in 2019; **(C)** The absolute number of DALYs in 21 regions in 1990 and 2019; **(D)** The EAPC of DALYs in 195 countries/territories in 2019.

Globally, the absolute number of DALYs increased by 95.71% (95% UI =82.15%–108.59%) from 1990 to 2019, reaching 24284.09 × 10^3^ (95% UI = 22614.92 × 10^3^ to 25723.22 × 10^3^) in 2019. Meanwhile, the ASDR decreased slightly from 1990 to 2019 (EAPC = -0.21, 95% UI = -0.26 to -0.15). In the 21 geographic regions, the absolute number of DALYs in 2019 was highest in East Asia (6712.86×10^3^, 95% UI = 5774.28×10^3^ to 7735.91×10^3^), and the largest increase in the number of DALYs, 264.23% (95% UI = 214.09%–322.09%), occurred in central Latin America (**Table 2**). In addition, the DALYs in Africa increased significantly, with an increase of 117.60% in North Africa and the Middle East. However, the DALYs in Western Europe increased by only 16.01% (95% UI = 11.24%–21.10%). Singapore and Austria had the greatest decline in trends of ASDR (EAPC less than -2.3) (**Figure 3**).

**Figure 4:**
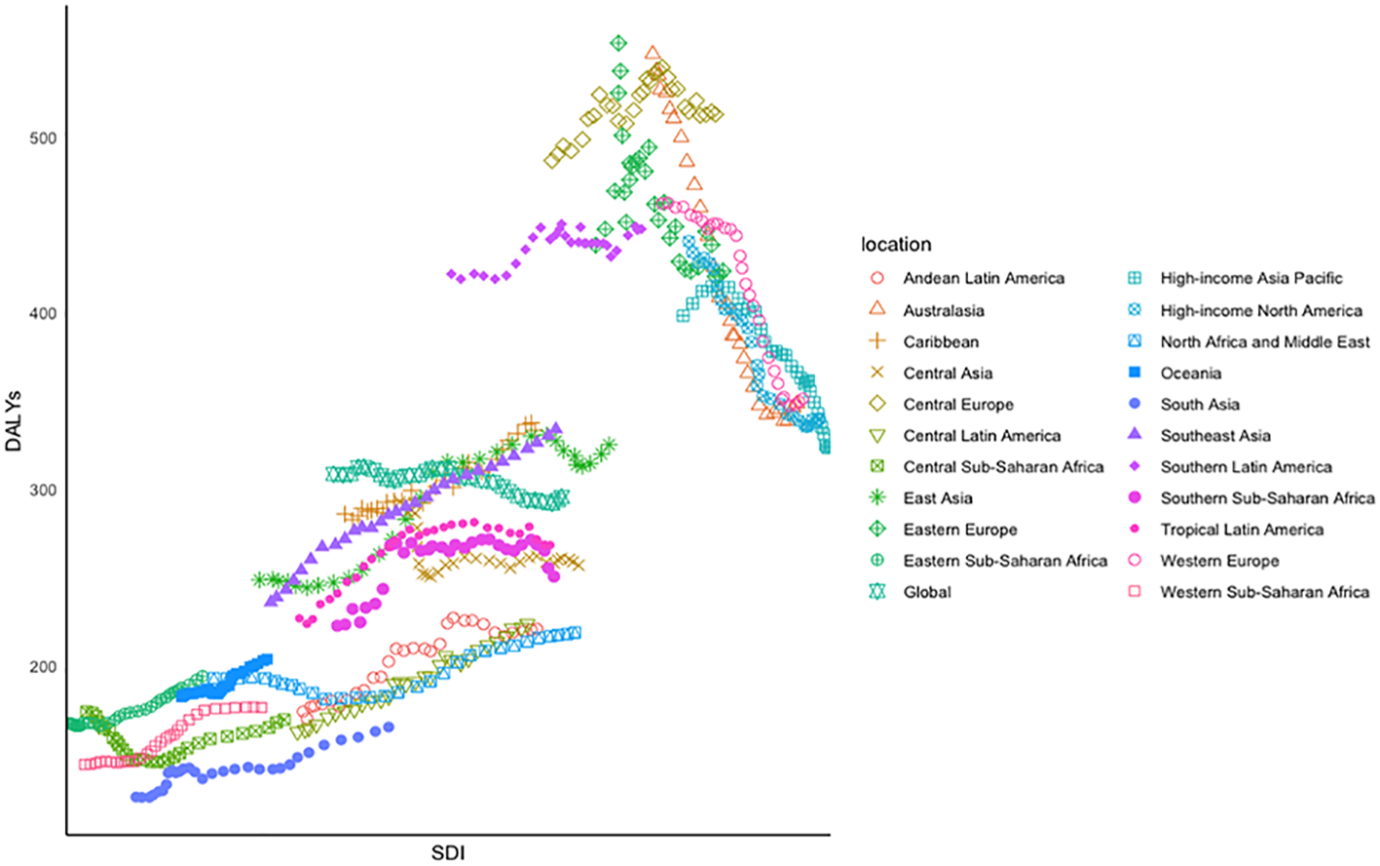
Age-standardized DALYs of CRC for 21 GBD regions by SDI between 1990 and 2019, in which different colors and shapes represent different regions.

Obvious polarization in the variation track of DALYs across the 21 GBD regions between 1990 and 2019 was observed (**Figure 4**). In the lower left corner of the figure, a relatively positive correlation was observed in regions such as South Asia, Southeast Asia, Western Europe, Western sub-Saharan Africa, and Eastern sub-Saharan Africa, where their ASDR increased with the fast-growing SDI. On the other hand, an inverse association was observed in Eastern Europe, Western Europe, and Australasia, where the SDI was growing slowly from 1990 to 2019, accompanied by rapidly declining ASDRs. Among the 21 SDI regions, the ASDR declined by 36.25% in Australasia and increased by 41.58% in Southeast Asia in 3 decades.

### The relationship between HDI and ASR

**Figure 5:**
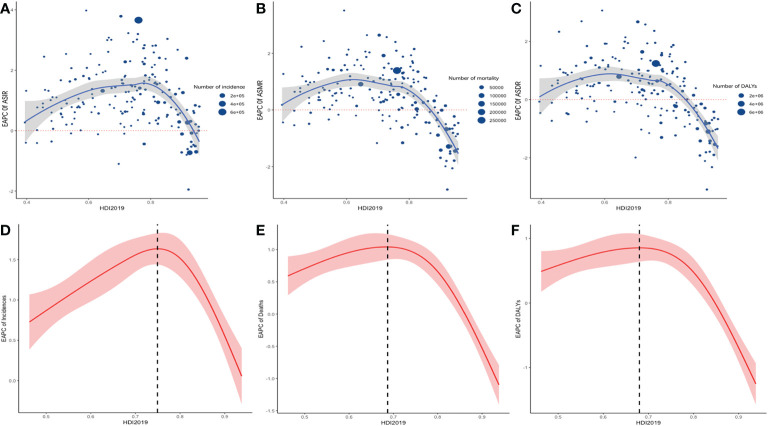
the relationship between HDI 2019 in 195 countries/territories and EAPC of ASR of incidence, mortality, and DALYs. Scatter plot and fitting curve described the corresponding relationship between HDI and EAPC of ASIR **(A)**, ASMR **(B)**, ASDR **(C)**, in which the size of the scatter represents the number of incidence **(A)**, mortality **(B)**, DALYs **(C)**. Display of nonlinear relationship between ASIR **(D)**, ASMR **(E)**, ASDR **(F)** and HDI 2019 in 195 countries/territories in restricted cubic spline models, shaded areas represent 95% CIs around incidence trend.

Pearson’s correlation analysis showed an exponential and positive correlation between ASIR and HDI (**Figure S1** in the supplementary material), with a correlation coefficient of 0.78. On the other hand, this study tested the overall and nonlinear association between the 2019 HDI and EAPC of ASR using RCS. The test for the overall association between the 2019 HDI and EAPC of ASR was significant, meaning that regardless of the shape of the association, 2019 HDI and EAPC were significantly correlated. In addition, the test of a nonlinear association was significant, indicating that the association was significantly nonlinear. The RCS curve on EAPC of ASIR and 2019 HDI showed an inverted V-shape, with the turning point at an HDI of 0.74 (**Figure 5**).

### The correlation analysis of meat consumption and ASIR

**Figure 6:**
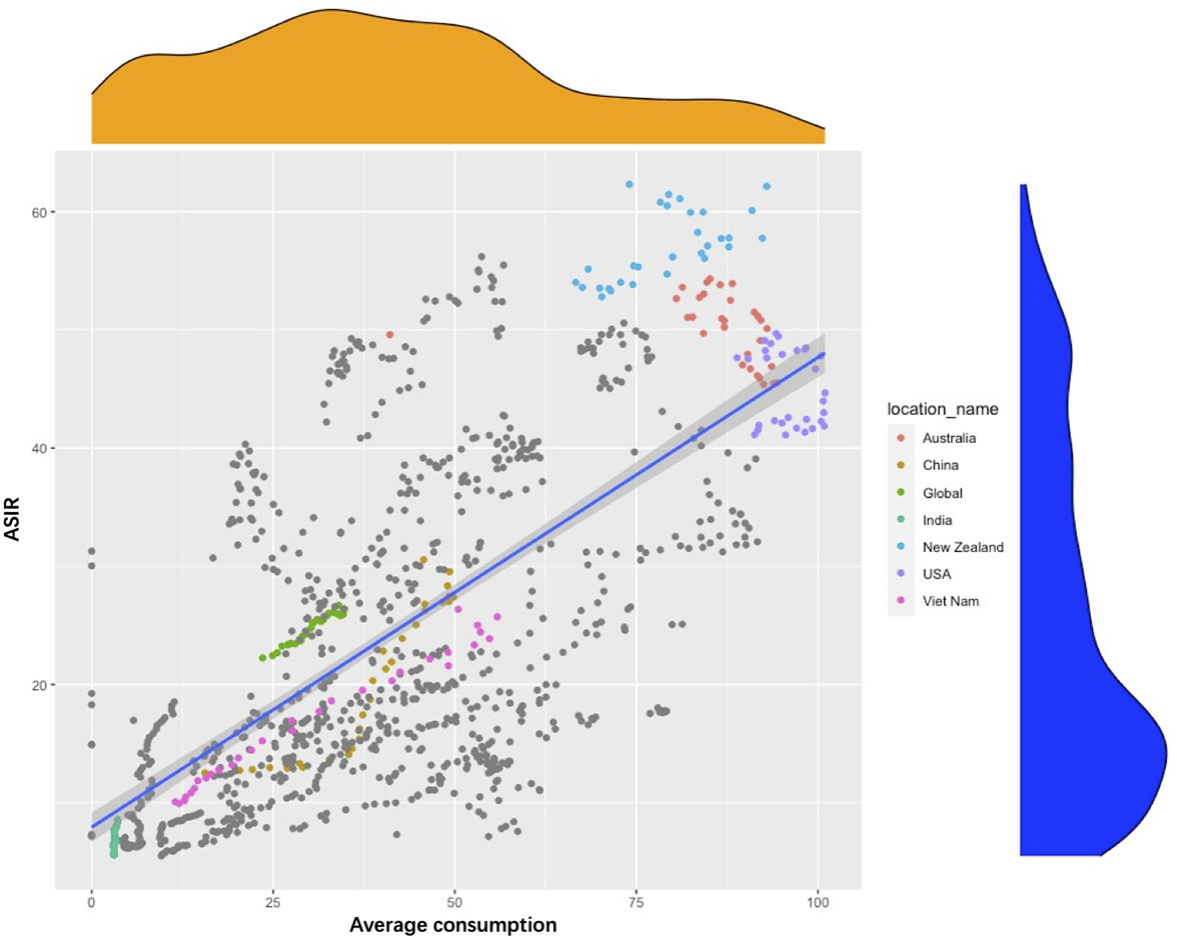
Scatter chart of the relationship between age-standardized rate of incidence and average meat consumption in selected countries around the world from 1990 to 2019.

To further investigate the relationship between dietary changes and ASIR, we included global data on the economic statistics of the nutrition and health industry-global meat consumption per capita (1990–2029) in this study. As shown in **Figure 6**, the relationship between meat consumption and ASIR showed a linear correlation in different countries from 1990 to 2019, especially for late-onset CRC in developing countries. In the United States, Australia, and New Zealand, meat consumption and the ASIR of CRC were both at high levels during the past 30 years, with little change and weak correlation. On the other hand, in China and Vietnam, meat consumption and ASIR showed a linear correlation and rapid increase during the past decades. In addition, India had a low-meat diet and low lower ASIR.

### Trends in the burden of CRC in different age groups

**Figure 7:**
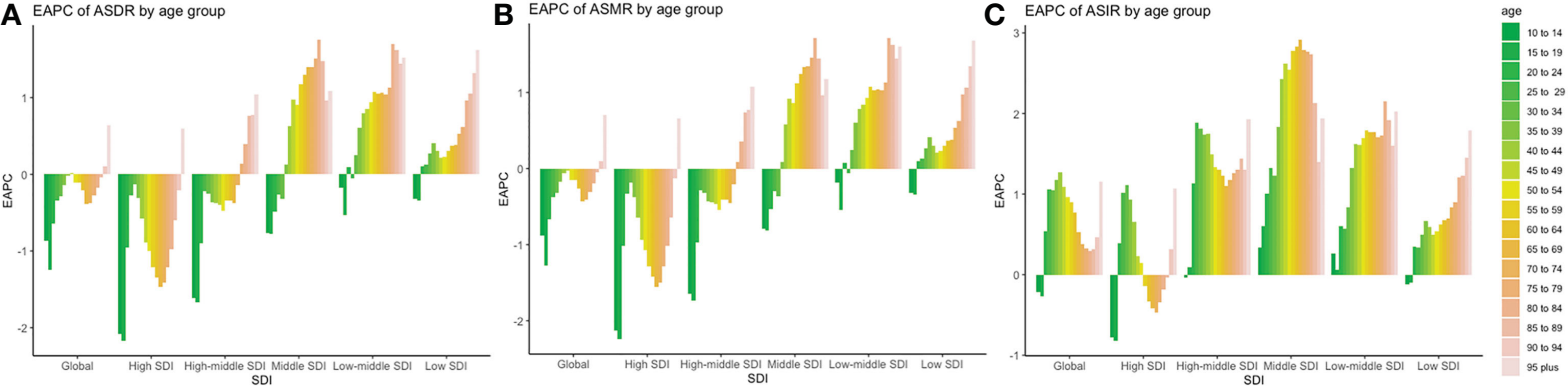
Trends of incidences, mortality, and DALYs in different age groups and SDI regions around the world. **(A)** EAPC of ASDR in different age and SDI groups; **(B)** EAPC of ASMR in different age and SDI group **(C)** EAPC of ASIR in different age and SDI groups.

Trends in ASR are shown in **Figure 7**. There were substantial differences in disease burden between different SDI regions. The ASIR showed an increasing trend in the global population over 20 years old, and the largest increasing trend occurred in the 25–49 age group (EAPC larger than 1.0), which illustrated that the incidence of early-onset CRC was in a stage of rapid growth than that of middle-aged and elderly population. Those in the 20–54 age group experienced an increasing trend in ASIR, while a downward trend was observed among those in the 55–90 age group. Among all age groups, the highest increasing trend occurred in the 30–34 age group (EAPC = 1.11), and the most obvious downward trend was observed in those aged 70–74 years old (EAPC = -0.47).

There was a steady increase of ASIR in high-middle SDI regions (EAPC >1), with two peaks among those in the 25–29 and 95-plus age groups (EAPC equal to 1.89 and 1.93, respectively). The fastest increase in the ASIR of early-onset CRC occurred in the 25–29 age group. In the middle SDI regions, the incidence increased steadily in all age groups, maintaining an abnormal increase trend in the 40–89 age group (EAPC >2.0). This histogram of EAPC was similar to a normal distribution, where EAPC peaked at the 65–69 age group (EAPC equal to 2.91). In the low-middle SDI regions, the fastest increase in trend was observed in the 80–84 age group (EAPC = 2.15). In the low-SDI areas, EAPC showed a steady increase by age, with a peak at the 95-plus age group (EAPC equal to 1.79).

The ASMR and ASDR showed the same downward trend globally, except for those over 90 years old. However, in the high SDI regions, the decreasing trend of ASMR and ASDR between the ages of 20 and 44 was slower compared to that of the 45–75 age group, especially in the age group of 30–34 (EAPC of ASMR = -0.18). In the high-middle SDI region, the ASMR and ASIR gradually decreased in age groups under 75 and increased among those over 75 years old. In the middle SDI region, ASMR and ASDR increased in those over 35 years old.

## The correlation analysis of antibiotic use and ASIR

**Figure 8:**
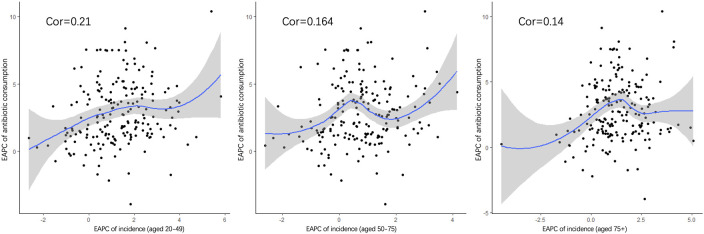
The correlation between the EAPC of ASIR in 20–49 **(A)**, 50–75, **(B)** and over 75 years old **(C)** and the annual percentage changes of antibiotic usage globally.

**Figure 9:**
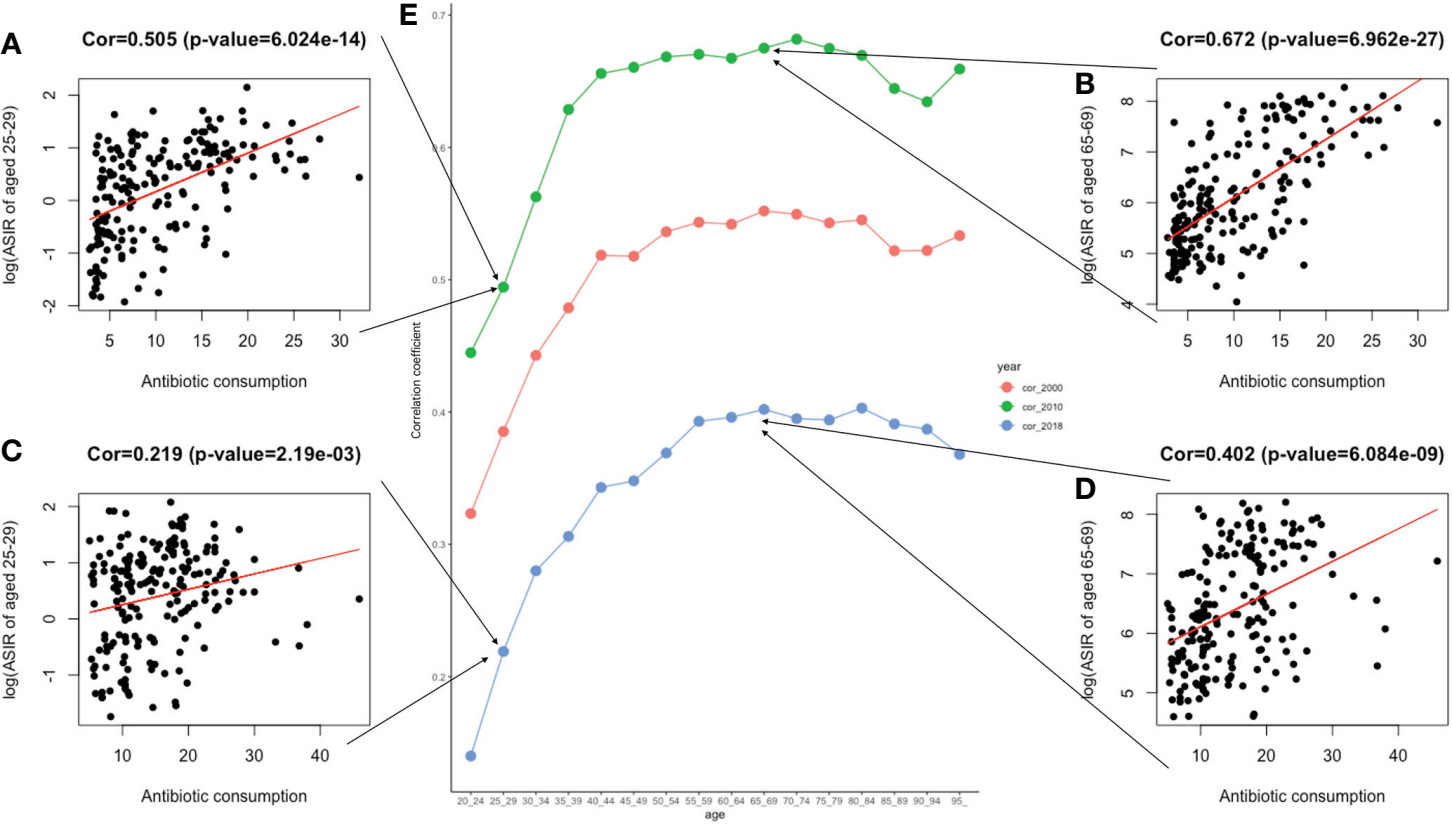
The correlation between antibiotic usage and ASIR in 2000, 2010, and 2018. **(A)**. The correlation analysis between ASIR and the global antibiotic usage in two age groups of 25–29 in 2010; **(B)**. The correlation analysis between ASIR and the global antibiotic usage in two age groups of 65–69 in 2010; **(C)**. The correlation analysis between ASIR and the global antibiotic usage in two age groups of 25–29 in 2018; **(D)**. The correlation analysis between ASIR and the global antibiotic usage in two age groups of 65–69 in 2018; **(E)**. Three lines graphs shown the value of correlation coefficient between ASIR of different age groups and global antimicrobial usage in 2000, 2010, and 2018.

Pearson’s correlation analysis of data on antibiotic use was conducted further explore the risk factors. **Figure 8** shows the correlation between the increasing trend of ASIR in those 20–49, 50–75, and over 75 years old and the EAPC of antibiotic use. The early-onset CRC group had the highest correlation (correlation coefficient = 0.21). In addition, we conducted a correlation analysis between ASIR and antibiotic use in different age groups. Similar correlation relationships were found in early-onset and late-onset CRC. In addition, the correlation coefficient of ASIR and antibiotic use increased with patients’ age in early-onset CRC but remained stable in late-onset CRC (**Figure 9**).

## Discussion

Although the age-standardized incidence, mortality, and DALYs of CRC have declined in some countries or regions around the world in the past 30 years, the absolute number has increased due to the development of global population growth, population aging, and the gradual increase of early-onset disease. Previous reports have shown that, to a certain extent, the incidence was related to the regional economy and seemed to be positively associated with the level of socioeconomic development (4, 20). This study reached similar conclusions that ASIR has increased exponentially with the growth of HDI. The reason why the RCS curve of 2019 HDI and ASIR showed an inverted V-shape might be that an increase in a country’s HDI leads to improvements in medical resources and medical insurance policy, resulting in amelioration of screening and medical treatment for diseases. This has brought about positive screening results of CRC over time, along with exponential increases in ASIR. However, after HDI increased to 0.74, the trend in ASIR remained stable or started declining. Globally, the incidence of CRC varied greatly across different regions, with decreasing trends in developed countries, represented by North America and Australia, and gradually increasing trends in developing countries, represented by South America and Southeast Asia. It is worth noting that the incidence in Asia was generally increasing, while the mortality rate within this region was quite different. In the past 3 decades, Vietnam experienced socioeconomic development, rapid urbanization, and lifestyle changes, and CRC was projected to be the most predominant cancer in Vietnamese males and the second most common cancer in females (21). However, the trend of ASDR and ASIR in China was significantly lower than in Vietnam. Such intra-regional differences could further be ascribed to socioeconomic development and improved healthcare resources, such as price negotiation on anti-cancer drugs, extensive medical insurance, and exempt tariffs on imported cancer drugs in China’s medical insurance policy (22, 23). In addition, the fastest decline in the trend of ASIR occurred in Austria, which was bound up with long-standing screening programs using colonoscopy and fecal tests (fecal test and colonoscopy screening programs were started in 1980 and 2005, respectively). About 68.6% of the population received chemical detection of fecal occult blood test (gFOBT) within 2 years or a colonoscopy within 10 years (24). The DALYs caused by CRC ranked third among the cancer-related causes of DALYs globally in 2019 (GBD 2019 Disease and Injury Incidence and Prevalence Collaborators, 2019), and the DALYs varied greatly on a global scale (3). In this study, the global imbalance in DALYs was manifested as an obvious two-level differentiation. The burden of CRC in low-SDI regions increased annually, largely due to the huge population and insufficient medical resources (25). Nevertheless, minor decreasing trends were observed in low and low-middle SDI regions, such as Southern sub-Saharan Africa, which might be an optimistic achievement of local health infrastructure, international cooperation, and health aid (25, 26).

On the other hand, ASDR has dropped by 36.26% in the past 30 years in Australasia, mainly due to The Australian National Bowel Cancer Screening Program fully rolled-out 2-yearly screening using the immunochemical Fecal Occult Blood Testing (iFOBT) in people aged 50–74 years (27). About 92,200 new cases and 59,000 deaths are estimated to be prevented from 2015 to 2040. Furthermore, a colorectal screening for people aged 55–59 years was included in the national bowel screening program in New Zealand, which significantly reduced the incidence and mortality of CRC (28). In Singapore, CRC screening included a fecal immunochemical test (FIT) performed annually, while a colonoscopy could be performed every 10 years. Moreover, since 2003, the community health assistance program under the census program has been implemented annually for citizens and permanent residents over the age of 50, and censuses on CRC were carried out with free screening kits provided by general practice clinics (29, 30). In addition, the recommended age for screening varies in different regions worldwide. Japan provides an annual FIT screening service for people aged 40 and over (31), which is one of the reasons for low mortality. Therefore, to reduce CRC mortality, the key lies in supporting a medical insurance system, public participation in colorectal cancer screening, and early screening.

Although the overall incidence of CRC in developed countries such as Europe and the United States was gradually decreasing, early-onset CRC now constitutes a substantial cancer burden among younger adults (32). The highest incidence of early-onset CRC occurred in the rectum, accounting for 42% (7). The incidence of colon cancer in adults aged 20–49 years increased by 6.4–9.3%, while that of rectal cancer increased by 1.6–3.5% annually in Europe (9). Our study found that the ASIR among the 25–34 age group in high SDI regions was fast-growing. This study suggests CRC in young adults should be monitored in future studies. The health administration departments in developing countries should not only popularize screening for CRC in middle-aged and elderly persons but also be alert to the prevalence of early-onset CRC. In addition, from a global perspective, the mortality rate of early-onset CRC patients in the 35–50 age group has decreased less than that of the middle-aged and elderly group, which was related to the more invasive molecular characteristics of early-onset CRC and high frequency of advanced stage at first diagnosis (33).

Although the causes of early-onset CRC remain unclear, earlier studies have explored disease pathogenesis, in which antibiotics use was considered a potential trigger (14), particularly in early-onset CRC (34). This study reached a similar conclusion to our work by showing that exposure to antibiotics might lead to CRC, especially in those under 50 years. In addition, the use of penicillin and anti-anaerobes in patients with CRC was higher than that in healthy people in a descriptive study. Another study showed that oral antibiotics, especially ampicillin/amoxicillin, increased the risk of colon cancer (OR = 1.09, 95% CI 1.05–1.13) (35). In this study, gradient differences in the association between the incidence of early-onset CRC and antimicrobial use in different age groups were found, possibly because the intestinal flora of patients under the age of 50 may show different changes under the stimulation of antibiotics. In addition, the correlation has gradually weakened in recent years, possibly due to the emergence of more factors that can interfere with bacterial diversity. Among the potential risk factors, obesity, enteritis, and diabetes need to be paid more attention. The global prevalence of age-standardized incidence of obesity increased from 0.7% in 1975 to 5.6% in 2016 (36), which may also be other latent risk factors for early-onset CRC (37). An environment of chronic tissue inflammation has been associated with malignant transformations, while diagnosing Crohn’s disease before 40 years old could increase the risk of CRC and even death (38). Diabetes increases the risk of CRC, especially early-onset CRC (39, 40). In addition, among diabetic patients, those not taking diabetes medications are more likely to develop adenomas than those taking medications (41). In our study, the risk proportion of fasting hyperglycemia has sharply increased by 41.8% globally in the past 3 decades (**Figure S2** in the supplementary material). Especially in high SDI regions, high blood glucose accounted for 9.6% of all risk factors, and there was a clear rise in trend in recent years. In addition, excess intake of high-fat foods containing a large amount of saturated fatty acids could easily lead to an imbalance in the gut microbiome, promoting the production of carcinogens and the occurrence of CRC (42). On the other hand, reduced exercise time and increased sedentary sitting affect telomere length and have profound effects on gut microbiota, which could increase the risk of CRC (43).

The incidence of late-onset CRC increased rapidly in developing countries, which was associated with local economic growth. With regional economic growth, diets have also undergone substantial changes. This study focused on the changing relationship between meat consumption and ASIR of CRC. In Asian developing countries, represented by China and Vietnam, with rapid economic growth in the last 30 years, meat consumption and the ASIR of CRC have shown rapid and synchronized growth. Contrastingly, in developed countries like the United States and Australia, meat consumption has not changed significantly in the past 3 decades with a steady HDI, and a weak correlation was found between CRC incidence and meat consumption in these regions. This finding shows that regional economic growth and improvements in HDI may significantly affect CRC incidence through changes in the dietary structure.

Moreover, studies have reported that a whole grain diet could reduce the risk of gastrointestinal cancer (44), and daily intake of dietary fiber up to 10 grams can reduce the risk of CRC by 10% (45). This is because phenolic compounds from microbial degradation from whole-grain diet catabolism can prevent CRC (46). In addition, evidence suggests that a daily intake of dietary calcium up to 200 mg can reduce the risk of CRC by 6% (42). Smoking and alcohol were still key risk factors, and their proportions were related to the implementation of tobacco and alcohol policies in different regions (**Supplementary Figure S3**). For example, Indonesia has the world’s largest tobacco market. The proportion of Indonesian men who smoked accounted for about 76.2% in 2015 and was still rising, leading to a sharp increase in the burden of tobacco-related diseases (47). As a result, anti-smoking policies were likely to effectively reduce the burden of CRC.

## Conclusion

In conclusion, this study found a geographical heterogeneity in the disease burden of CRC. The HDI in 2019 was exponentially and positively correlated with ASIR in all countries, and HDI was nonlinearly associated with the EAPC of ASR, demonstrating an inverted V-shaped relationship. Affected by HDI, changes in dietary patterns, such as meat consumption, were influenced by the economic level and strongly correlated with the incidence of CRC. In addition, antibiotic use has a potential effect on the occurrence of CRC, especially for early-onset CRC. Trends in the ASIR on early-onset CRC across different age groups, especially for young adults in high- and high-middle SDI regions, showed a fast-growing trend. Meanwhile, the mortality and DALYs of late-onset CRC were growing fast in the middle and middle-low SDI regions.

Therefore, CRC prevention programs in developing countries should not only popularize screening and control the consumption of tobacco and alcohol but also strictly control meat consumption and antibiotic use. Specifically, countries in low SDI regions should rationally regulate the use of antibiotics, reduce the abuse of antibiotics, strengthen the control of the tobacco and alcohol consumption market, and popularize screening for middle-aged and elderly persons. Meanwhile, developed countries should advocate for self-testing with fecal occult blood kits and hospital visits among young people, especially those at high risk for early-onset CRC.

## Strengths and limitations of this study

The present study used the 2019 GBD database to describe the incidence, mortality, DALYs, and risk factors for CRC in various countries and regions worldwide. The limitations of this study are mainly related to the limitations of the 2019 GBD database itself, which is generated using an algorithm based on existing data in every country and depends largely on the quality and quantity of data. Therefore, the data of countries at low levels of development were scarce and of low quality, which is a significant limitation. In addition, due to the lag in data reporting, our estimates may not include recent changes in health data across regions. Disease risk factors are numerous and unclear, especially early-onset colon cancer. This study did not explore all the risk factors for CRC, which require further research.

## Data availability statement

The datasets presented in this study can be found in online repositories. The names of the repository/repositories and accession number(s) can be found in the article/**Supplementary Material**.

## Author contributions

Project development: S-QF and Z-YM. Data collection: Y-HZ and Y-XL. Data analysis: W-XS and X-BS. Manuscript writing/editing: L-BL and L-YW. Language polishing and manuscript revision: D-MC. All authors contributed to the article and approved the submitted version.
