# Supplementary material for: A systematic analysis of the global and regional burden of colon and rectum cancer and the difference between early- and late-onset CRC from 1990 to 2019
Source: Front Oncol. 2023 Feb 15;13:1102673. doi: 10.3389/fonc.2023.1102673 (PMC9975717; doi:10.3389/fonc.2023.1102673)
Supplement: Supplementary file 1 [file DataSheet_1.zip › supplementary material/supplementary material figures/supplementary material.docx]

Figure s1: The correlation analysis and fitting curve between HDI 2019 and ASIR, and the correlation coefficient equal to 0.78. The best fitting model was as follows:y=144.58x^2−129.17x+36.678 (Adjusted R2=0.66, F-statistic=183.13, and the p value is less than 2.22e-16)

Figure s2: The proportion of CRC DALYs attributable to seven risk for 21 GBD regions, 2019BMI=body-mass index. DALY=disability-adjusted life-year. GBD=Global Burden of Diseases, Injuries, and Risk Factors Study.

Figure s3: The proportion of seven main risk factors of DALYs from 1999 to 2019. BMI: Body-mass index
