# Supplementary figures and images for: A systematic analysis of the global and regional burden of colon and rectum cancer and the difference between early- and late-onset CRC from 1990 to 2019
Source: Front Oncol. 2023 Feb 15;13:1102673. doi: 10.3389/fonc.2023.1102673 (PMC9975717; doi:10.3389/fonc.2023.1102673)

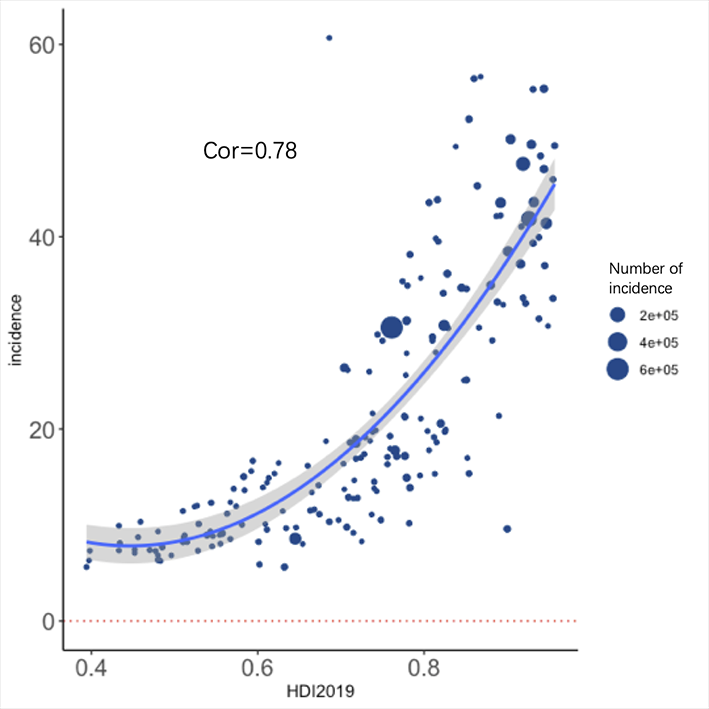

Supplement: Supplementary file 1 [file DataSheet_1.zip › supplementary material/supplementary material figures/F s1.tiff]

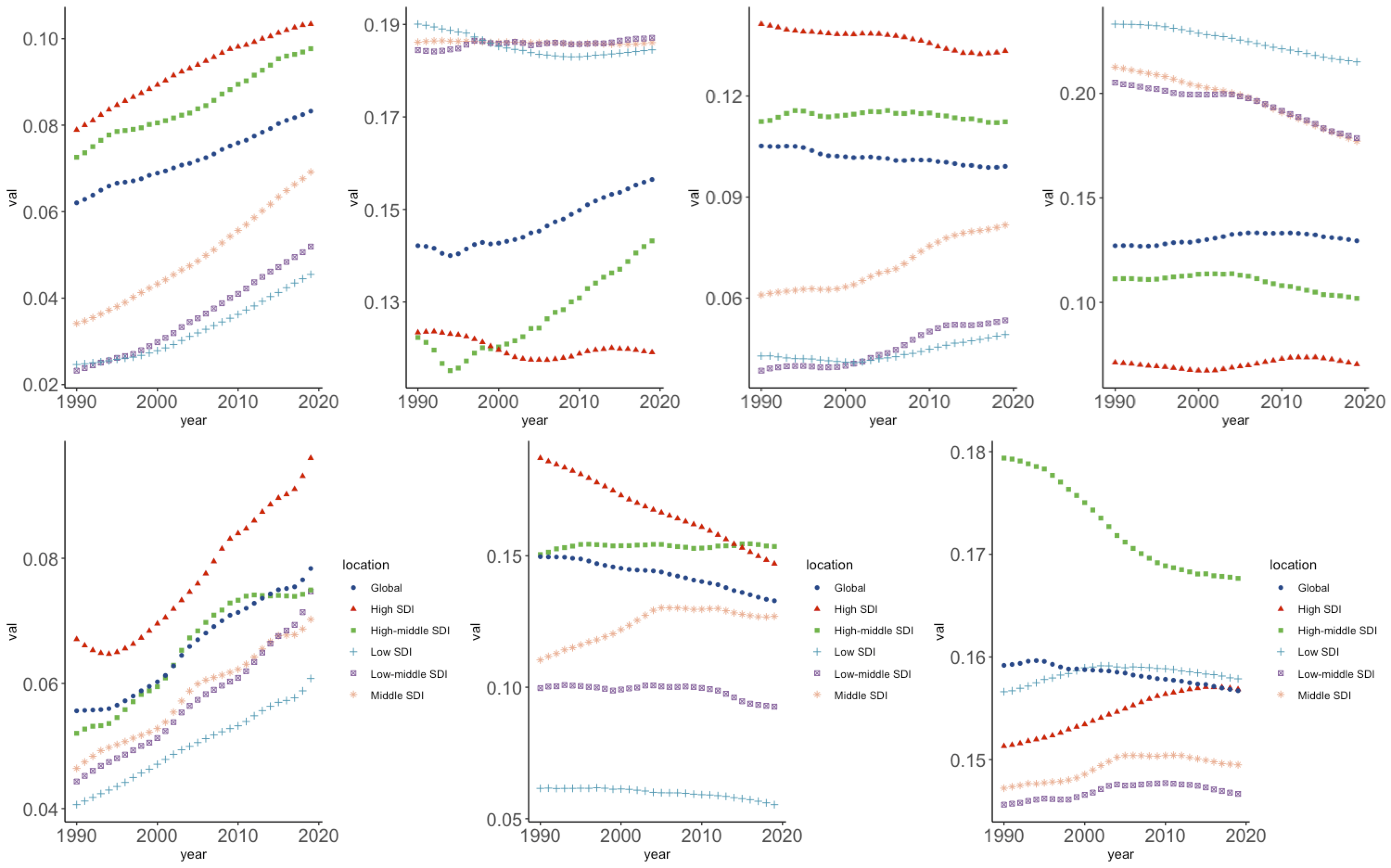

Supplement: Supplementary file 1 [file DataSheet_1.zip › supplementary material/supplementary material figures/F s3.tiff]

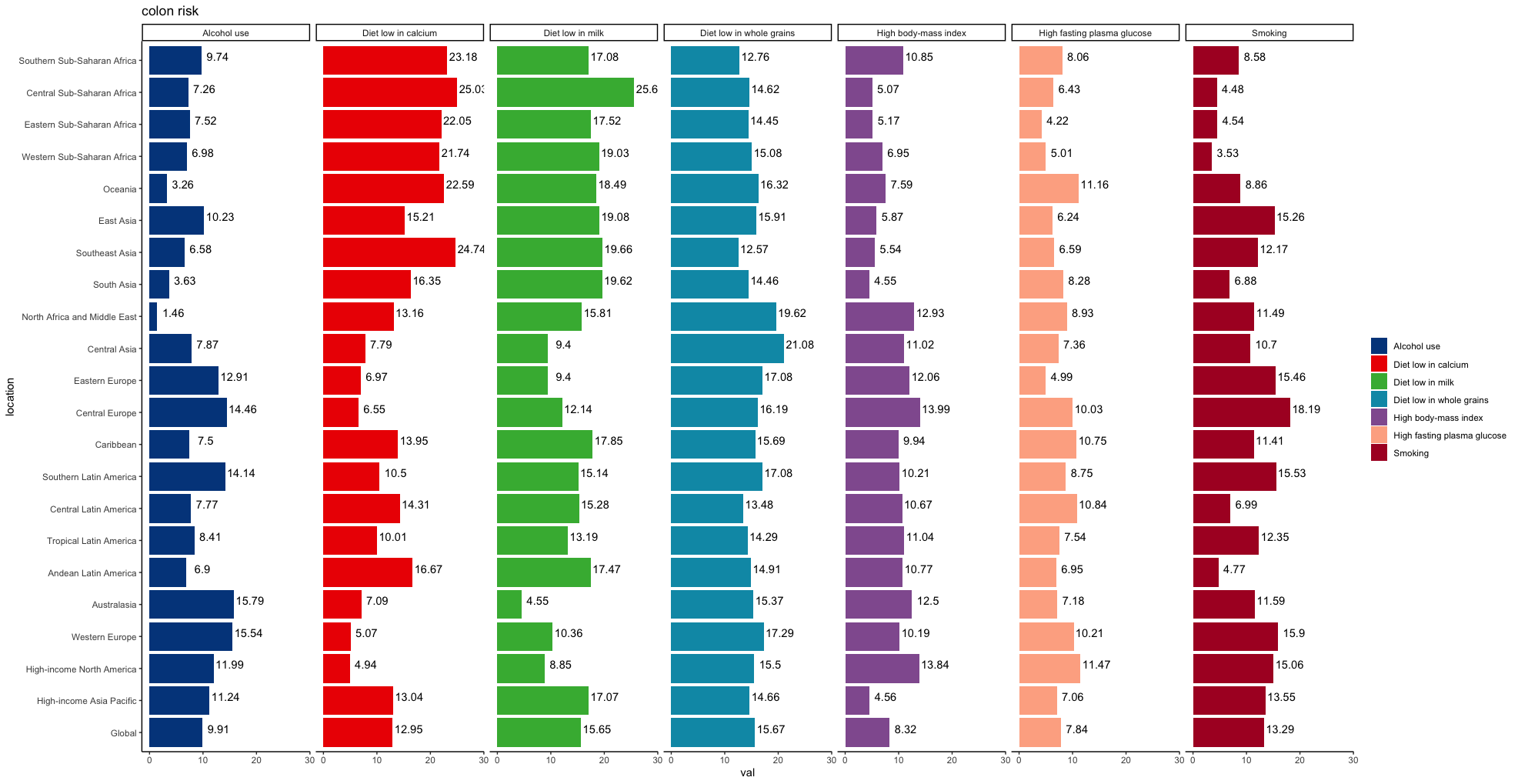

Supplement: Supplementary file 1 [file DataSheet_1.zip › supplementary material/supplementary material figures/F s2.tiff]
